# Modeling Huntington’s disease against age-related genes reveals *CXXC4* as an epigenetic target to restore DRD1 striatal neuron activity

**DOI:** 10.64898/2025.12.12.693965

**Published:** 2026-01-13

**Authors:** Maialen Arrieta-Lobo, Francesca Farina, Tamara Monteagudo Aboy, Megan Mair, Cloé Mendoza, Huy Tran, Jeff Aaronson, Jim Rosinski, Lisa Ellerby, Emmanuel Brouillet, Jean-Michel Peyrin, Juan Botas, Christian Neri, Lucile Megret

## Abstract

Neurons adapt gene expression to counter aging, yet the mechanisms by which they harness age- related genes to resist neurodegenerative disease remain elusive. We found that transcriptionalaging inversion (TAGI) in the *Drd1* -expressing striatal neurons ( *Drd1* SNs) of Huntington’s disease (HD) knock-in ( *Hdh* ) mice exhibits discrete patterns against a TAG-like (TAGL) signature. Strikingly, TAGI dynamics may explain disease progression more accurately than TAGL. Moreover, in *Drd1* SNs, genes affected by 3’UTR accumulation during aging are predisposed to downregulation in aging and deregulation in *Hdh* mice. By integrating age-related 3’UTR data and *Hdh* -mice data, we identified a CAG-repeat-dependent network of upregulated genes with compensatory potential. This network features (i) *Atad-5,* a P CNA unloader that modifies CAG expansion in human HD plasma samples, and (ii) *CXXC4* (IDAX), an epigenetic regulator whose early-stage upregulation is lost as behavioral symptoms worsen in *Hdh* mice. Functionally, *CXXC4* reduces the senescence marker p16INK4a and restores glutamate excitability in human HD iPS cell-derived SNs. Collectively, these findings suggest that *Drd1* SN resilience capacity against HD relies on discrete age-related patterns and responses transiently activated in early disease stages. The reactivation of specific age-related genes such as *CXXC4* may notably restore cortico-striatal function in HD.

## Full Text Availability

The license terms selected by the author(s) for this preprint version do not permit archiving in PMC. The full text is available from the preprint server.

