# Supplementary material for: Modeling the effects of Huntington’s disease on age-related genes reveals CXXC4 as an epigenetic target to restore health and excitability of Drd1-expressing striatal neurons
Source: bioRxiv. 2025 Dec 15:2025.12.12.693965. Preprint. [Version 1] doi: 10.64898/2025.12.12.693965 (PMC12724660; doi:10.64898/2025.12.12.693965)
Supplement: Supplementary file 1 [file NIHPP2025.12.12.693965v1-supplement-3.pdf]

SUPPLEMENTARY INFORMATION

Supplementary Tables

Supplementary Table 1. Differential expression analysis of HD and normal-aging TRAP-seq data in mouse *Drd1*-expressing striatal neurons, gene set category (aging-like and aging-inversion effects), *Geomic* v 1.1 assignation, temporal profile, whole-striatum LFC changes between 2 months and 6 months, and whole-striatum LFC changes between 6 months and 10 months.

Supplementary Table 2. Probabilistic overlap analyzes between aging-like or aging-inversion effects in the *Drd1*-expressing striatal neurons of *Hdh* mice and datasets of interest.

bioRxiv preprint doi: https://doi.org/10.64898/2025.12.12.693965; this version posted December 15, 2025. The copyright holder for this preprint (which was not certified by peer review) is the author/funder, who has granted bioRxiv a license to display the preprint in perpetuity. It is made available under aCC-BY 4.0 International license.

Supplementary Table 3. Probabilistic overlap analyzes between deregulated genes (TRAP-seq data) and genes affected by 3'UTR accumulation in the *Drd1*-expressing striatal neurons of 24-month-old wild-type mice[16].

Supplementary Table 4. Probabilistic overlap analyzes between genes deregulated in *Hdh* mice (TRAP-seq data)[14], genes deregulated in aging (TRAP-seq data)[16], and genes genes affected by 3'UTR accumulation in aging[16], all datasets from the *Drd1*-expressing striatal neurons.

Supplementary Table 5. Connectivity matrices for Rando-SDS networks defining Class-1 and Class-2 genes for CAG repeat size Q50, Q111 and merged Q(170-175).

Supplementary Table 6. Information for Class-1 and Class-2 genes, including gene-expression levels at different CAG repeat lengths, R_{tc} values representing the 3'UTR accumulation, *Geomic* v 1.1 assignation, temporal profile, bulk striatum LFC changes between 2 and 6 months, bulk striatum LFC changes between 6 and 10 months, and CS-1 and CS-2 scores.

Supplementary Table 7. Class-1 and Class-2 genes for Q111 and Q(170–175), with and without inclusion of first-neighbour interactors.

Supplementary Table 8. Overlaps between Class-1 or Class-2 genes and genes in datasets of interest.

Supplementary Table 9. Functional annotations for Class-1 or Class-2 gene nodes and their neighbours as indicated by *Geomic* analysis[6] and by functional shRNA screening in *Hdh* mice[21].

Supplementary Table 10. Overview of *Drosophila* screen data, including ortholog mapping, genotype/allele information, observed phenotypes, and statistical significance of genotype, time, and type of effects.

bioRxiv preprint doi: https://doi.org/10.64898/2025.12.12.693965; this version posted December 15, 2025. The copyright holder for this preprint (which was not certified by peer review) is the author/funder, who has granted bioRxiv a license to display the preprint in perpetuity. It is made available under aCC-BY 4.0 International license.

Supplementary figures

Supplementary Figure 1 related to Figure 3. (**a**) Machine learning pipeline for characterizing cumulative effect class (CEC) and non-cumulative effect class (non-CEC) signatures in the *Drd1* SNs of *Hdh* mice. The analysis pipeline takes advantage of two datasets, the first corresponding to RNA-seq data from the *Drd1* SNs from 6-month-old *Hdh* mice bearing 4 CAG-repeat lengths, and the second to 3'UTR accumulation data from the *Drd1* SNs of 24-month-old wild type mice. In a first step, using statistical and bionetwork-based approaches for each CAG-repeat length, the pipeline classifies genes retained by the model into two different groups: genes subjected to down regulation in HD and 3'UTR accumulation in normal aging and genes subjected to up regulation in HD and 3'UTR accumulation in normal aging. In a second step, applying an improved version (v. 1.1) of *Geomic* analysis[6], the pipeline performs gene-by-gene inference of temporal dynamics of CEC and non-CEC effects by exploiting whole striatum RNA-seq data of *Hdh* mice mice at 2 months, 6 months and 10 months of age, assigning the temporal information that is available in the whole striatum data to CAG-repeat dependent data of *Drd1* SNs. (**b**) Interconnections between the Q111 Class-1 and Class-2 networks in the *Drd-1* SNs of *Hdh* mice at 6-months. Class-1 nodes are represented in dark red, Class-2 nodes in dark green, nodes downregulated in HD but not present in the Class 1 signature are light red, while downregulated in HD but not present in the Class 2 signature are light green. Node size is proportional to the CS1 and CS2 scores of Class-1 and Class-2 genes, respectively. Most remarkable EnrichR annotations are also indicated. (**c**) Temporal dynamics of Class-1 and Class-2 effects in the *Drd1* SNs of *Hdh*-Q111 mice. Out of 389 genes retained by our model in the combined Q(170-175) network (246 Class-1 genes and 143 Class-2 genes), 222 genes (168 Class-1 genes and 54 Class-2 genes) were assigned at least to the *Drd1* SNs. These genes are further classified into 4 temporal subclasses: reduced Class-1 effects, maintained Class-1 effects, reduced Class-2 effects and maintained Class-2 effects. Panels feature 2D representations of each of the four classes, where the x-axis corresponds to the difference between LFC values at and at 6 months compared to 2 months (ΔLFC_{6M-2M}) and y-axis to the difference between LFC values 10 months compared to 6 months (ΔLFC_{10M-6M}): (top left) Class-1 genes whose temporal profile is reduced across age points [39 genes]; (top right) Class-2 genes whose temporal profile is reduced across age points [3 genes]; (bottom left) Class-1 genes whose temporal profile is maintained age points [11 genes] and (bottom right) Class-2 genes whose

temporal profile is maintained across age points [2 genes]. The straight line in each panel represents the y = x line where the two ΔLFC have the same value. The color gradient represents the combined Q(170-175) CS-1 or CS-2 score at 6 months of age. For each panel, a number of interesting genes have been highlighted.

Supplementary Figure 2 related to Figure 6. Quality control of MSN differentiation and lentiviral transduction. (**a**) Representative immunofluorescence images of human iPS cell-derived MSNs C116 (CAG-corrected: 21Q/19Q) and HD (72Q/19Q) stained for gamma-aminobutyric acid (GABA) and DARPP32. Scale bar: 30 µm. Right panel shows the percentage of cells positive for each marker. Data are mean±SD. (**b**) Upper panels show qPCR analysis of relative mRNA expression levels of *RFP* and *CXXC4* in transduced cells compared to untreated cells (UT). Lower panel shows the percentage of MAP2- and RFP-positive cells based on immunofluorescence analysis, indicating successful neuronal differentiation and effective transduction. Data are mean±SD. (**c**) Quantification of nuclear p16INK4a intensity per cell in unrelated HD-60Q and control-18Q iPS cell lines (18Qn2 LV tRFP: n = 184 cells, 18Qn2 LV CXXC4-tRFP: n = 126 cells, HD60Qn8 LV tRFP: n = 121 cells, HD60Qn8 LV CXXC4-tRFP: n = 59 cells). Experiment indicating that *CXXC4* expression may reduce nuclear p16INK4a levels in both human HD60Qn8 and control 18Qn2 iPS cell-derived MSNs. Paired *t*-test, $^{****}P < 0.0001$.
